# Hemispheric Asymmetry of Functional Brain Networks under Different Emotions Using EEG Data

**DOI:** 10.3390/e22090939

**Published:** 2020-08-26

**Authors:** Rui Cao, Huiyu Shi, Xin Wang, Shoujun Huo, Yan Hao, Bin Wang, Hao Guo, Jie Xiang

**Affiliations:** 1 Department of Software Engineering, College of Software, Taiyuan University of Technology, Taiyuan 030600, China; shihuiyu0833@link.tyut.edu.cn (H.S.); shoujunhuo@gmail.com (S.H.); haoyan0832@link.tyut.edu.cn (Y.H.); 2 Department of Computer Science and Technology, College of Information and Computer, Taiyuan University of Technology, Taiyuan 030600, China; wangxin0735@link.tyut.edu.cn (X.W.); wangbin01@tyut.edu.cn (B.W.); guohao@tyut.edu.cn (H.G.); xiangjie@tyut.edu.cn (J.X.)

**Keywords:** hemispheric asymmetry, graph theory, brain network, emotion

## Abstract

Despite many studies reporting hemispheric asymmetry in the representation and processing of emotions, the essence of the asymmetry remains controversial. Brain network analysis based on electroencephalography (EEG) is a useful biological method to study brain function. Here, EEG data were recorded while participants watched different emotional videos. According to the videos’ emotional categories, the data were divided into four categories: high arousal high valence (HAHV), low arousal high valence (LAHV), low arousal low valence (LALV) and high arousal low valence (HALV). The phase lag index as a connectivity index was calculated in theta (4–7 Hz), alpha (8–13 Hz), beta (14–30 Hz) and gamma (31–45 Hz) bands. Hemispheric networks were constructed for each trial, and graph theory was applied to quantify the hemispheric networks’ topological properties. Statistical analyses showed significant topological differences in the gamma band. The left hemispheric network showed significantly higher clustering coefficient (*C_p_*), global efficiency (*E_g_*) and local efficiency (*E_loc_*) and lower characteristic path length (*L_p_*) under HAHV emotion. The right hemispheric network showed significantly higher *C_p_* and *E_loc_* and lower *L_p_* under HALV emotion. The results showed that the left hemisphere was dominant for HAHV emotion, while the right hemisphere was dominant for HALV emotion. The research revealed the relationship between emotion and hemispheric asymmetry from the perspective of brain networks.

## 1. Introduction

Emotions refer to various affective state of human beings arising as a response to some interpersonal or other events [1] and play an important role in interpersonal communication [2]. Emotions in humans are subjective conscious experiences characterized by psycho-physiological responses, and people have different heart rates, blood pressure, skin conductance responses and brain activities under different emotional states [3]. Among these psycho-physiological responses, asymmetrical brain activity has received increasing attention in recent years. However, the essence of hemispheric asymmetry during emotion processing remains controversial. Expressions of happiness and fear were identified faster when presented in the left visual field, suggesting an advantage of the right hemisphere in the perception of these expressions [4]. In contrast, some researchers found that right and left hemisphere responses varied as a function of emotional types, with increased right hemisphere activity for negative emotions and an increased left hemisphere response for positive emotions [5,6,7]. Two asymmetric hypotheses can be used to explain the opposite experimental results. The first hypotheses, labeled “the right hemisphere hypothesis”, posits that the right hemisphere is dominant for processing all emotions regardless of affective valence [8,9]. The second hypotheses, named “the valence hypothesis”, assumes that the right hemisphere is specialized for negative emotions, while the left hemisphere is specialized for positive emotions [10,11].

In the past, traditional methods of electroencephalography (EEG) analysis, including alpha power [12], amplitude [13] and coherence [14], were widely used in studies of hemispheric asymmetry during different emotional states. During emotional processing, mutual connections exist between various brain regions, which function synergistically. However, the traditional EEG analysis method cannot reveal these connections. Brain network analysis based on EEG is a useful biological method that reflects brain function [15,16]. At the same time, graph theory is a powerful mathematical tool that allows analysis and quantification of brain networks [17,18]. Studies have proven that some more interesting results can be revealed through brain networks than through traditional EEG analysis methods [19,20]. Previous studies have rarely examined hemispheric differences in the topological organization of brain networks during emotion processing. Therefore, this study aimed to reveal the association between emotion and hemispheric asymmetry from the perspective of functional brain networks.

## 2. Materials and Methods

### 2.1. EEG Data Acquisition

The Database for Emotional Analysis using Physiological Signals (DEAP) [2] is a multimodal dataset for the analysis of human affective states. The DEAP dataset contains physiological recordings from 32 healthy subjects, with 50% males and 50% females, aged between 19 and 37 with a mean age of 26.9 years. The EEG data were collected while participants watched 40 one-minute music videos corresponding to different emotional genres.

### 2.2. Data Preprocessing

**Figure 1:**
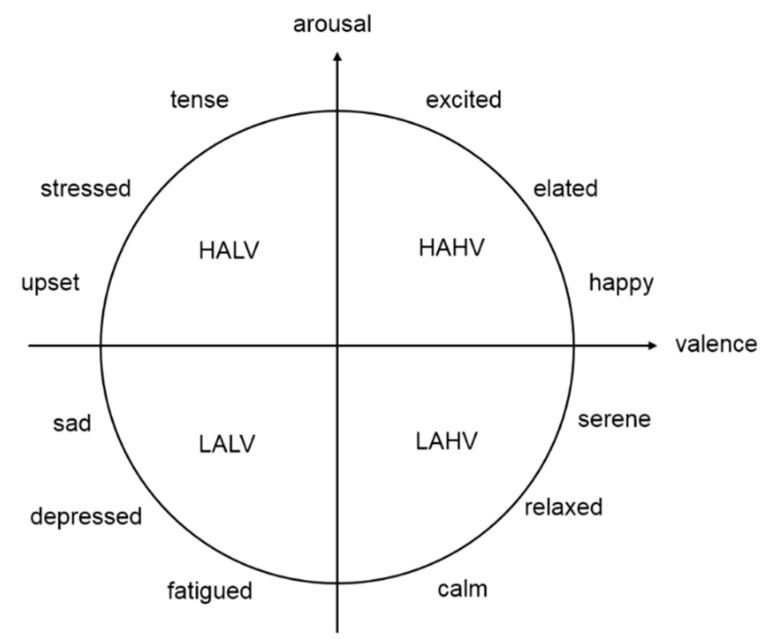
Arousal–valence plane. The valence dimension evaluates the degree of positivity or negativity of an emotion, and the arousal dimension describes the intensity of activation associated with an emotion.

The raw EEG data were preprocessed in a series of steps, including down-sampling to 128 Hz, removing electro-oculography (EOG) artifacts with a blind source separation technique that can recover a set of independent source signals from linear mixtures of them [21], filtering each channel to 4–45 Hz and averaging channels to the common reference. For each participant, 40 music videos were presented in 40 trials. After each trial, their ratings (ranging from 1 to 9) of arousal (A) and valence (V) for the music videos presented were recorded. V ranged from unpleasant to pleasant, and A ranged from inactive to active. A threshold of 5 is a common choice in this type of analysis and has been used in similar studies previously [22,23]. Therefore, based on A and V, we selected a rating of 5 as the threshold to divided the emotions into four categories: high valence (HAHV), low arousal high valence (LAHV), low arousal low valence (LALV) and high arousal low valence (HALV), as shown in Figure 1. According to these different emotional states, the EEG data were divided into four categories. In the rating process, the EEG data of subject 23 were only divided into three categories. Therefore, the EEG data recorded from subject 23 was discarded, and the EEG data of the remaining subjects were retained. The number of EEG data in each category is shown in Table 1.

### 2.3. Frequency Division

In the human brain, different oscillation frequencies of neurons are closely related to the functional activities of the brain [24]. Therefore, we use the finite impulse response (FIR) filter in the EEGLAB toolbox [25] to divide the EEG data into four frequency bands: θ (4–7 Hz), α (8–13 Hz), β (14–30 Hz) and γ (31–45 Hz).

### 2.4. Network Construction

Nodes and edges are the two basic elements of a network. In this study, scalp electrodes were used as nodes of the network, and phase lag index (PLI) was used for the network edge to measure the relationship between two nodes. In the past, coherence [26], synchronization likelihood [27] and other indicators were used to express the strength of relationships between nodes. However, coherence and synchronization likelihood are highly affected by volume conduction, and coherence in particular is also affected by the time series amplitude [28]. The volume conduction in EEG results from the mixing of signals of neural origin caused by the conductive properties of the environment through which the signals propagate [28]. The major purpose of introducing the PLI is to obtain a reliable estimate of phase synchronization that is invariant against the presence of common sources [29]. The PLI is a measure of the asymmetry in the distribution of phase differences between two signals, which can reflect the consistency of one signal’s phase leading or lagging relative to another signal. To compute the PLI, the instantaneous phase must be estimated using an analytic signal method. The analytical signal $x^{H}\;$ is complex-valued with x(t), a real time series, and $\tilde{x}\left(t\right)$, its Hilbert transform:

(1) $$x^{H}\left(t\right)=x\left(t\right)+i\tilde{x}\left(t\right)=A\left(t\right)e^{i\phi \left(t\right)}$$

The instantaneous amplitude A(t) and the instantaneous phase ϕ(t) can be computed by:

(2) $$A\left(t\right)=\sqrt{{\left[\tilde{x}\left(t\right)\right]}^{2}+{\left[x\left(t\right)\right]}^{2}}$$

(3) $$\phi \left(t\right)=arctan\frac{\tilde{x}\left(t\right)}{x\left(t\right)}$$

The PLI can be obtained from phase differences $∆\phi \left(t_{k}\right)$, k = 1 … *N* in the following equation:

(4) $$\Delta \phi \left(t_{k}\right)=\phi_{1}\left(t_{k}\right)-\phi_{2}\left(t_{k}\right)$$

(5) $$PLI=\left|\langle sign\left[sin\left(∆\phi \left(t_{k}\right)\right)\right]\rangle \right|$$

The $\phi_{1}\left(t_{k}\right)$ and $\phi_{2}\left(t_{k}\right)$ are the phases of two time series. The “sign” reflects the signum function. The PLI ranges from 0 to 1. A value of 0 indicates no coupling or coupling with a phase difference centered around 0 mod π, while a value of 1 indicates perfect phase locking at a value of ∆ϕ different from 0 mod π [29].

**Figure 2:**
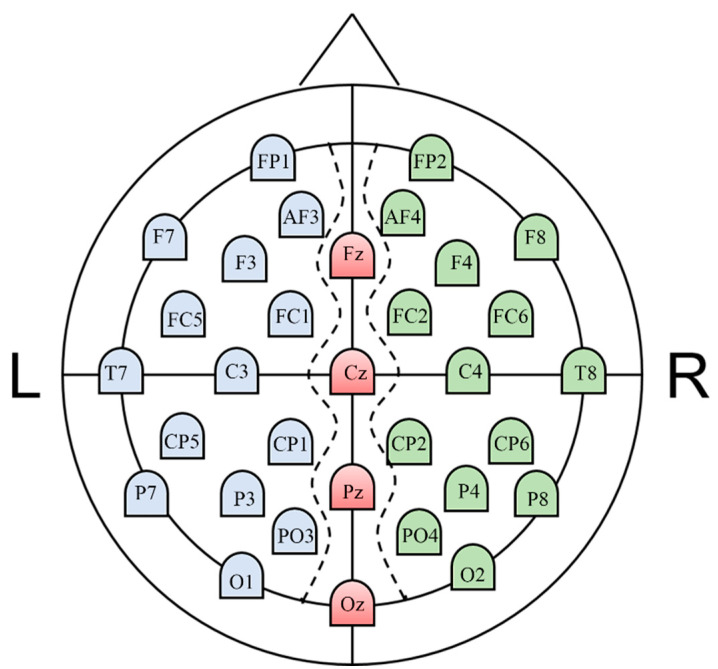
Detailed electrode positions used in the Database for Emotional Analysis using Physiological Signals (DEAP) database.

As shown in Figure 2, we used 18 nodes (red and blue areas) in the left hemisphere to construct the left hemispheric network. Similarly, 18 nodes (red and green areas) in the right hemisphere were used to construct the right hemispheric network. For each trial, we eliminated the interhemispheric connections and then obtained two weighted 18 × 18 hemispheric brain networks, including one for the left hemisphere and another for the right hemisphere [30]. We implemented GRETNA software [31] to investigate the network properties of each trial. Before calculating network properties, we applied a sparsity threshold (S_thr_), which retains S_thr_% of the top connections for each network. To avoid biases related to using a single threshold, we investigated network properties within a range of 5% < S_thr_ < 40% in steps of 1% because previous studies have shown that networks are small-world within this range [32]. In the next step, we calculated the area under the curve (AUC) for each network property across the full range of sparsity thresholds. Additionally, we averaged the AUCs of multiple trials of the same subject in each group.

### 2.5. Network Analysis

At the global and regional levels, this study investigated six network properties to analyze the topological organization of the networks. The five global properties included clustering coefficient (*C_p_*), characteristic path length (*L_p_*), small-world property (*σ*), global efficiency (*E_g_*) and local efficiency (*E_loc_*). C_p_ of a node is computed as the number of connections that exist between the nearest neighbors of this node as a proportion of the maximum number of possible connections. The network *C_p_* is the average of *C_p_* of all nodes in the network and indicates the strength of the local interconnection of a given network. A high *C_p_* indicates that nodes tend to form dense regional cliques, implying high efficiency in local information transfer [33]. *L_p_* is the average shortest path length between all pairs of nodes in the network. A low *L_p_* ensures rapid transmission of information in the whole network. A healthy human brain maintains two different properties: functional segregation and functional integration [34]. Functional segregation indicates locally specialized information processing in the brain, and functional integration indicates both information integration from distributed brain regions and global efficiency of information transfer [35,36]. *C_p_* and *L_p_* are respectively functional segregation and functional integration measures in static brain networks [37,38]. Watts and Strogatz proposed a small-world network model which has a high *C_p_* similar to a regular network but a low *L_p_* similar to a random network [32]. The σ of a network can be characterized by both the normalized *C_p_* and the normalized *L_p_*, indicating a balance between functional integration and segregation. E_g_ is a method to measure the efficiency of distant information transmission in the network and is defined as the inverse of the average shortest path length between all pairs of nodes in the network [39,40]. *E_loc_* of a node is the inverse of the average shortest path length of all neighbors of this node among themselves. A network *E_loc_* is the average of *E_loc_* of all nodes in the network and is a measure of the average efficiency of information transfer within local subnetworks [39,40]. The regional property is described in term of nodal efficiency (*E_nodal_*), which measures the information transmission ability of node *i* in the network [40]. Higher nodal efficiency indicates strong interconnectivity with other nodes. The definitions and brief interpretations of the network properties are shown in Table 2.

### 2.6. Asymmetry Score

The asymmetry score (AS) [41] of the above-mentioned network properties was calculated according to the following formulation:

(6) AS(X)=(X(R)−X(L))/(X(R)+X(L))×100

where *X(L)* and *X(R)* represent network properties of the left and right hemispheres, respectively. The AS helped us to analyze differences between the left and right hemispheres. *AS(X)* ranges from −100 to +100. A positive value of *AS(X)* represents rightward asymmetry, while a negative value of *AS(X)* indicates leftward asymmetry.

## 3. Results

### 3.1. Global Properties of Hemispheric Networks

**Figure 3:**
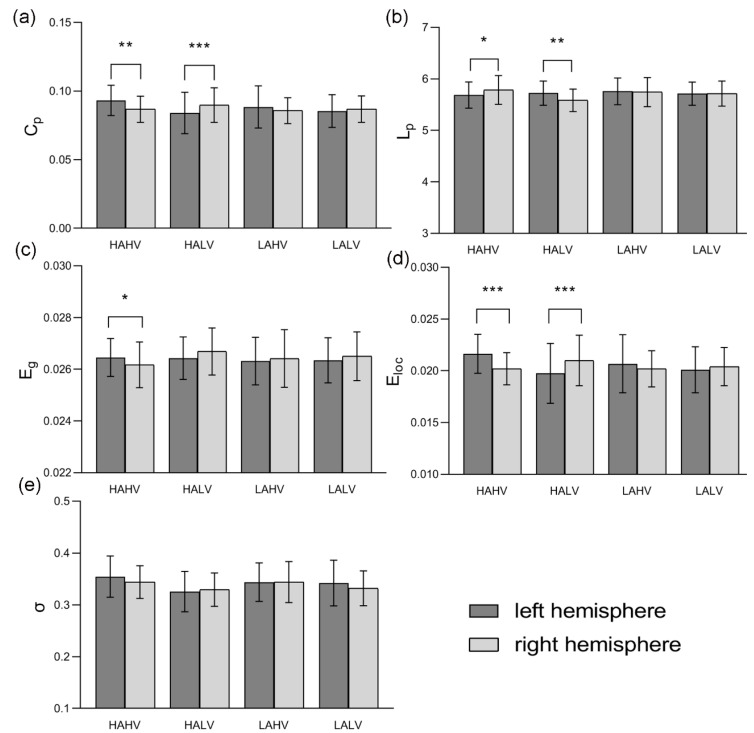
Changes in the values of (**a**) *C_p_*, (**b**) *L_p_*, (**c**) *E_g_*, (**d**) *E_loc_*, and (**e**) σ between the left and right hemispheric networks under the four different categories of emotions (HAHV, HALV, LAHV and LALV). Bars represent mean ± standard deviation. Significant differences are marked with asterisks: * *p* < 0.05, ** *p* < 0.01, *** *p* < 0.001.

In this study, left and right hemispheric networks were constructed for each trial under HAHV, HALV, LAHV and LALV emotions. A paired t-test was used to evaluate the significance of differences between the left and right hemispheres within each group. The data were verified to conform to a normal distribution before the *t*-test was applied. Figures S1–S3 of the Supplementary Materials show differences between the left and right hemispheres in theta, alpha, and beta bands, respectively. Since no significant hemispherical differences were found in these bands, we used these results as Supplementary Materials. As shown in Figure 3, statistical analyses showed significant topological differences between left and right hemispheric networks in the gamma band under HAHV and HALV emotions. Every network property exhibited significant hemispheric differences except for *σ* under HAHV emotion. Specifically, the left hemispheric network showed significantly higher clustering coefficient *C_p_* (*t* = 3.520, *p* = 0.001), global efficiency *E_g_* (*t* = 2.387, *p* = 0.024) and local efficiency *E_loc_* (*t* = 5.282, *p* = 0.000) and significantly lower characteristic path length *L_p_* (*t* = −2.133, *p* = 0.041) than the right hemispheric network under HAHV emotion. The above results indicate that nodes in left hemisphere tend to form dense regional cliques, implying high efficiency in information transfer under HAHV emotion. Furthermore, the right hemispheric network showed significantly higher clustering coefficient *C_p_* (*t* = −4.622, *p* = 0.000) and local efficiency *E_loc_* (*t* = −3.905, *p* = 0.000) and significantly lower characteristic path length *L_p_* (*t* = 2.897, *p* = 0.007) than the left hemispheric network, suggesting that activation in the right hemisphere was higher than that in the left hemisphere. In addition, no significant differences were observed between the two hemispheric networks under LAHV and LALV emotions.

### 3.2. Asymmetry Scores of Global Properties

ASs can sensitively reflect differences in network properties between the left hemisphere and right hemisphere. Statistical analyses of ASs of the global properties are shown in Table 3. Significant differences in hemispheric asymmetry were observed only under HAHV and HALV emotions. The left hemispheric networks tended to be more globally efficient (negative AS (*E_g_*), *p* = 0.022) and locally efficient (negative AS (*E_loc_*), *p* = 0.000) under HAHV emotion. Additionally, the clustering coefficient (negative AS (*C_p_*), *p* = 0.001) showed leftward hemispheric asymmetry, and the characteristic path length (positive AS (*L_p_*), *p* = 0.037) showed rightward hemispheric asymmetry. Under HALV emotion, the right hemispheric networks tended to be more locally efficient (positive AS (*E_loc_*), *p* = 0.001). Meanwhile, the clustering coefficient (positive AS (*C_p_*), *p* = 0.000) showed rightward hemispheric asymmetry, and the characteristic path length (negative AS (*L_p_*), *p* = 0.007) showed leftward hemispheric asymmetry. These results indicate that hemispheric asymmetry changes with different emotions.

### 3.3. Regional Properties of Hemispheric Networks

**Figure 4:**
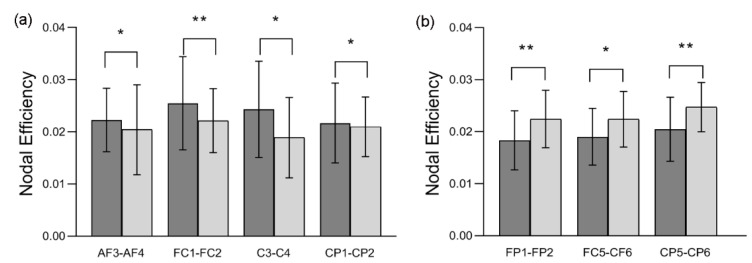
Nodal efficiency differences in corresponding nodes in the left and right hemispheres. Significant differences are marked with asterisks: * *p* < 0.05, ** *p* < 0.01, *** *p* < 0.001 (paired *t*-test for hemispheric differences).

**Figure 5:**
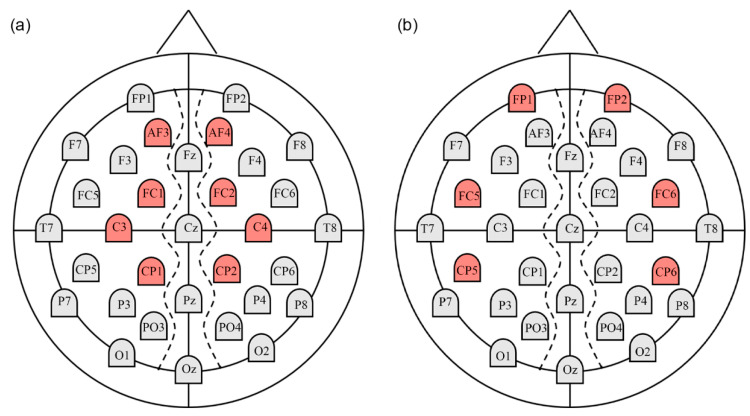
The locations of the electrodes with significant differences in nodal efficiency under (**a**) HAHV and (**b**) HALV emotions (the electrodes marked in red represent those with significant differences).

The statistical results for nodal efficiency differences are summarized in Figure 4. Using false discovery rate (FDR) correction, we observed that nodal efficiency exhibited significant hemispheric differences (*p* < 0.05) between the two hemispheres under HAHV and HALV emotion. The node pairs with a significant leftward advantage in nodal efficiency mainly included AF3-AF4 (*t* = 2.217, *p* = 0.034), FC1-FC2 (*t* = 2.782, *p* = 0.009), C3-C4 (*t* = 2.243, *p* = 0.032) and CP1-CP2 (*t* = 2.255, *p* = 0.032) under HAHV emotion, as shown in Figure 4a. The locations of the four node pairs are shown in Figure 5a. These results show that information transmission efficiency is higher in the left hemispheric nodes during HAHV emotion. Under HALV emotion, node pairs with a significant rightward advantage in nodal efficiency mainly included FP1-FP2 (*t* = −2.899, *p* = 0.007), FC5-FC6 (*t* = −2.599, *p* = 0.014), and CP5-CP6 (*t* = −3.230, *p* = 0.003), as shown in Figure 4b, and the locations of the three node pairs are shown in Figure 5b, which indicate that the information transmission efficiency of the right hemispheric nodes is higher under HALV emotion.

### 3.4. Asymmetry Score of Nodal Efficiency

**Figure 6:**
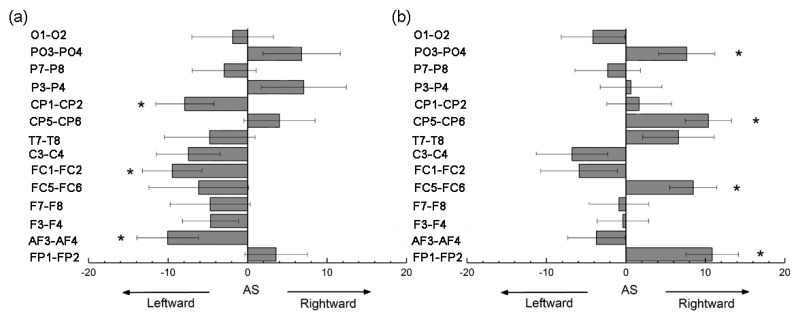
The asymmetry scores (ASs) for nodal efficiency in the hemispheric networks under (**a**) HAHV and (**b**) HALV emotions. Each bar indicates the mean AS. Error bars represent the standard error. For both HAHV and HALV emotions, node pairs with significant ASs are marked with asterisks (* *p* < 0.05).

The statistical results for the AS (*E_nodal_*) are summarized in Figure 6. Significant (*p* < 0.05, FDR-corrected) asymmetries were observed in three node pairs in the hemispheric networks of HAHV and four node pairs in the hemispheric networks of HALV. As shown in Figure 6a, AF3-AF4 (negative AS (*E_nodal_*), *t* = −2.585, *p* = 0.015), FC1-FC2 (negative AS (*E_nodal_*), *t* = −2.533, *p* = 0.017) and CP1-CP2 (negative AS (*E_nodal_*), *t* = −2.162, *p* = 0.039) showed significant leftward hemispheric asymmetries under HAHV emotion. As shown in Figure 6b, FP1-FP2 (positive AS (*E_nodal_*), *t* = 3.283, *p* = 0.003), FC5-FC6 (positive AS (*E_nodal_*), *t* = 2.858, *p* = 0.008), CP5-CP6 (positive AS (*E_nodal_*), *t* = 3.573, *p* = 0.001) and PO3-PO4 (positive AS (*E_nodal_*), *t* = 2.151, *p* = 0.040) showed significant rightward hemispheric asymmetries under HALV emotion.

## 4. Discussion

In this study, we used graph theoretical approaches to investigate the hemispheric asymmetry of functional brain networks under different emotions. Differences between left and right hemispheric networks were mainly found in the gamma band. The reason for the above results is that the gamma band is highly sensitive to emotional states. Serap used an asymmetry measurement method in EEG sub-bands (delta, theta, alpha, beta, and gamma) and found that inter-hemispheric emotional functions are mostly mediated by the gamma band [42].

As shown in Figure 3, the left hemispheric network showed significantly higher *C_p_*, *E_g_* and *E_loc_* and lower *L_p_* than the right hemispheric network under HAHV emotion. The high *C_p_* indicates that nodes tend to form dense regional cliques, demonstrating high efficiency of local information transmission [33]. A low *L_p_* ensures prompt transfer of global information which is a measure of functional integration [43]. Under HAHV emotion, nodes in the left hemisphere tend to form dense regional cliques, and the left hemispheric network was more clustered compared with the right hemispheric network. This network topology caused the *E_g_* and *E_loc_* of the left hemispheric network to be higher than those of the right hemispheric network. The above results indicated that activation in the left hemisphere was higher than that in the right hemisphere, and that the left hemisphere was closely related to HAHV emotion. Similar to our results, the study by Guozhen Zhao observed greater left hemispheric activation when subjects were watching a positive film [7]. Under HALV emotion, *C_p_* and *E_loc_* were significantly higher, while *L_p_* was lower in the right hemispheric networks than those in the left hemispheric networks, indicating that the high density of connections between functionally related regions in the right hemisphere increases the clustering coefficient of the network. Compared with the left hemispheric network, the right hemispheric network had more long-range connections between different nodes, resulting in a low characteristic path length. Our study showed that the activation of the right hemisphere was higher than that of the left hemisphere during HALV emotion, indicating that the right hemisphere was closely related to HALV emotion [44]. These results clearly support the valence hypothesis. No significant differences were found between the left and right hemispheric networks under LAHV and LALV emotions. One possible reason for this result is that both hemispheres were less active when subjects’ emotional states were characterized by low arousal. Previous studies have similarly found that high arousal increased the brain’s gamma activity compared with low arousal [45]. Miskovic found that viewing highly arousing images led to significant enhancement of EEG coherence between prefrontal and posterior electrodes in both cerebral hemispheres [46]. In this study, we found that both hemispheric networks under HAHV, HALV, LAHV and LALV emotions are small-world networks; therefore, each group showed no significant differences in small-world property. Bassett provided strong new evidence for the existence of functional networks exhibiting small-world property in the human brain [47]. However, few reports are available on whether hemispheric networks exhibit small-world property.

Statistical analyses of the ASs for the global properties are shown in Table 3. Under HAHV emotion, we found that *C_p_*, *E_g_* and *E_loc_* showed significant leftward asymmetry, while *L_p_* showed significant rightward asymmetry. Significant rightward asymmetries in *C_p_* and *E_loc_*, while significant leftward asymmetries in *L_p_* were observed under HALV emotion. These results further suggest that hemispheric asymmetry varied with different emotions. We can conclude that the left hemisphere is dominant for HAHV emotion, and that the right hemisphere is dominant for HALV emotion. The above results further support the valence hypothesis over the right hemisphere hypothesis. The right hemisphere hypothesis was proposed by Gainotti on the basis of clinical observations of patients with right and left hemisphere lesions [48]. However, all participants in our experiment were normal subjects. The results may be due to differences in brain activity between normal subjects and patients when processing emotions.

As shown in Figure 4, under HAHV emotion, we found significant leftward asymmetries in nodal efficiency in several node pairs (including AF3-AF4, FC1-FC2, C3-C4, and CP1-CP2). Meanwhile, under HALV emotion, significant rightward asymmetries in nodal efficiency were observed in several node pairs (including FP1-FP2, FC5-FC6 and CP5-CP6). Nodal efficiency measures the information transmission efficiency of nodes in a network [40]. A node with high nodal efficiency indicates strong interconnectivity with other nodes in the network. The analysis results show that information transmission efficiency was higher in the left hemispheric nodes than in the right hemispheric nodes during HAHV emotion. However, the information transmission efficiency of right hemispheric nodes was higher than that of left hemispheric nodes under HALV emotion. From the local properties of brain networks, the results also supported the valence hypothesis but opposed the right hemisphere hypothesis. Interestingly, we found that the node pairs with significant differences were not the same under HAHV and HALV emotions, but they were mainly concentrated in the frontal and parietal areas, suggesting that these areas may be closely related to emotional processing. Previous studies have also shown that the frontal and parietal regions play an important role in emotional processing [42,49]. For nodal efficiency, we found that the ASs for AF3-AF4, FC1-FC2 and CP1-CP2 were negative under HAHV emotion, while the ASs for FP1-FP2, FC5-CF6, CP5-CP6 and PO3-PO4 were positive under HALV emotion. The results showed that the left hemisphere was more activated than the right hemisphere during the processing of HAHV emotion, while the right hemisphere was more activated than the left hemisphere under HALV emotion.

There may be some possible limitations in this study. We used PLI to measure the relationship between nodes and tried to use the weighted phase lag index (wPLI) as connectivity index. The results of the two methods are similar, so we do not report the results of wPLI in this paper for brevity. The reason for the above results may be that PLI and wPLI can reduce the volume conduction effect, but the volume conduction effect may still have an impact on the research results. In the follow-up study, we try to use EEG source localization to reveal the cortical sources underlying brain activity, which can effectively solve the above problems.

## 5. Conclusions

This study used graph theory to analyze global and regional properties of left and right hemispheric networks under different emotions. Activation was found to be significantly increased in the right hemisphere during the processing of HALV emotion, while activation in the left hemisphere increased significantly when processing HAHV emotion. This research concluded that the right hemisphere was specialized for HALV emotions, and that the left hemisphere was specialized for HAHV emotions. At the same time, we found that the frontal and parietal areas are closely related to emotional processing by comparing the nodal efficiency of the left and right hemispheric networks. This research revealed the relationship between emotion and hemispheric asymmetry from the perspective of functional brain networks, which supported the valence hypothesis.

## Figures and Tables

**Table 1:** Number of electroencephalography (EEG) data per emotion category.

Category	HAHV	HALV	LAHV	LALV
Number of EEG data	429	298	253	260

**Table 2:** Formulations and descriptions of network topological properties applied in this work.

Network Properties	Definitions	Measurement and Interpretation
Clustering coefficient (*C_p_*)	$C_{p}=\frac{1}{N}\underset{i\epsilon G}{\sum }\frac{E_{i}}{D_{i}\left(D_{i}-1\right)/2}$	$D_{i}$ is the number of neighbors of node *i,* and $E_{i}$ is the number of edges between neighbors of node *i*. *N* is the number of nodes in the network *G* (*N* = 18 in this work).
Characteristic path length (*L_p_*)	$L_{p}=\frac{1}{N\left(N-1\right)}\underset{i\neq j\epsilon G}{\sum }L_{i,j}$	$L_{i,j}\;$ is the shortest path length between nodes *i* and *j*.
Global efficiency (*E_g_*)	$E_{g}=\frac{1}{N\left(N-1\right)}\underset{i\neq j\epsilon G}{\sum }\frac{1}{L_{i,j}}$	$E_{g}$ reflects the information transmission efficiency of the whole network.
Local efficiency (*E_loc_*)	$E_{loc}=\frac{1}{N}\underset{i\epsilon G}{\sum }E_{loc}\left(i\right)$	$E_{loc}\left(i\right)$ is local efficiency of node i, and $E_{loc}$ is a measure of the average local efficiency of network.
Small-world property (*σ*)	$\sigma =\frac{C_{p}/C_{random}}{L_{p}/L_{random}}$	$C_{p}/C_{random}$ represents the normalized clustering coefficient.$\;L_{p}/L_{random}$ represents the normalized characteristic path length.
Nodal efficiency (*E_nodal_*)	$E_{nodal}\left(i\right)=\frac{1}{N\left(N-1\right)}\underset{j\epsilon G}{\sum }\frac{1}{L_{i,j}}$	$E_{nodal}\left(i\right)$ measures the information transmission ability of node *i* in network *G*.

**Table 3:** Differences in the asymmetry scores of the global properties.

Properties	HAHV Mean (*p*-Value)	HALV Mean (*p*-Value)	LAHV Mean (*p*-Value)	LALV Mean (*p*-Value)
AS (*C_p_*)	−3.565 (**0.001**)	3.668 (**<0.001**)	−2.239 (0.204)	0.983 (0.270)
AS (*L_p_*)	0.850 (**0.037**)	−1.234 (**0.007**)	−0.131 (0.735)	−0.002 (0.996)
AS (*E_g_*)	−0.549 (**0.022**)	0.485 (0.138)	0.174 (0.542)	0.291 (0.338)
AS (*E_loc_*)	−3.386 (**<0.001**)	3.266 (**0.001**)	−1.789 (0.771)	0.861 (0.306)
AS (*σ*)	−1.369 (0.244)	1.076 (0.470)	−0.447 (0.725)	−1.316 (0.378)

A one-sample two-tailed *t*-test was used to evaluate the statistical results within each group. A significant effect (*p* < 0.05) of a network property is expressed in bold.

## Supplementary Materials

The following are available at: https://www.mdpi.com/1099-4300/22/9/939/s1. Figure S1: Changes in the values of (a) *C_p_*, (b) *L_p_*, (c) *E_g_*, (d) *E_loc_*, and (e) σ between the left and right hemispheric networks under the four different categories of emotions in theta band. Bars represent mean ± standard deviation. Figure S2: Changes in the values of (a) *C_p_*, (b) *L_p_*, (c) *E_g_*, (d) *E_loc_*, and (e) σ between the left and right hemispheric networks under the four different categories of emotions in alpha band. Bars represent mean ± standard deviation. Figure S3: Changes in the values of (a) *C_p_*, (b) *L_p_*, (c) *E_g_*, (d) *E_loc_*, and (e) σ between the left and right hemispheric networks under the four different categories of emotions in beta band. Bars represent mean ± standard deviation.
