# Supplementary figures and images for: Hemispheric Asymmetry of Functional Brain Networks under Different Emotions Using EEG Data
Source: Entropy (Basel). 2020 Aug 26;22(9):939. doi: 10.3390/e22090939 (PMC7597206; doi:10.3390/e22090939)

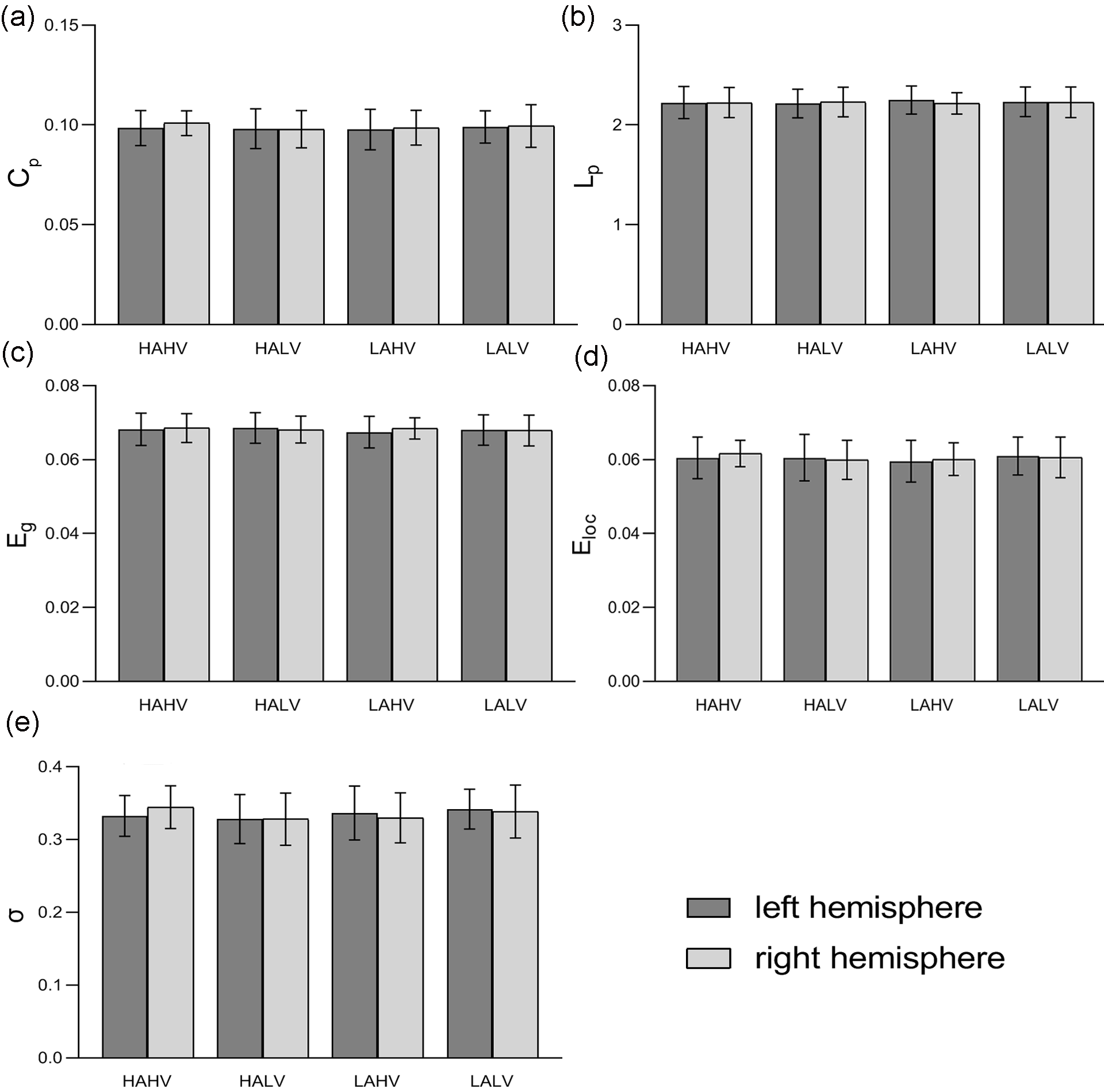

Supplement: Supplementary file 1 [file entropy-22-00939-s001.zip › Figure S1.tif]

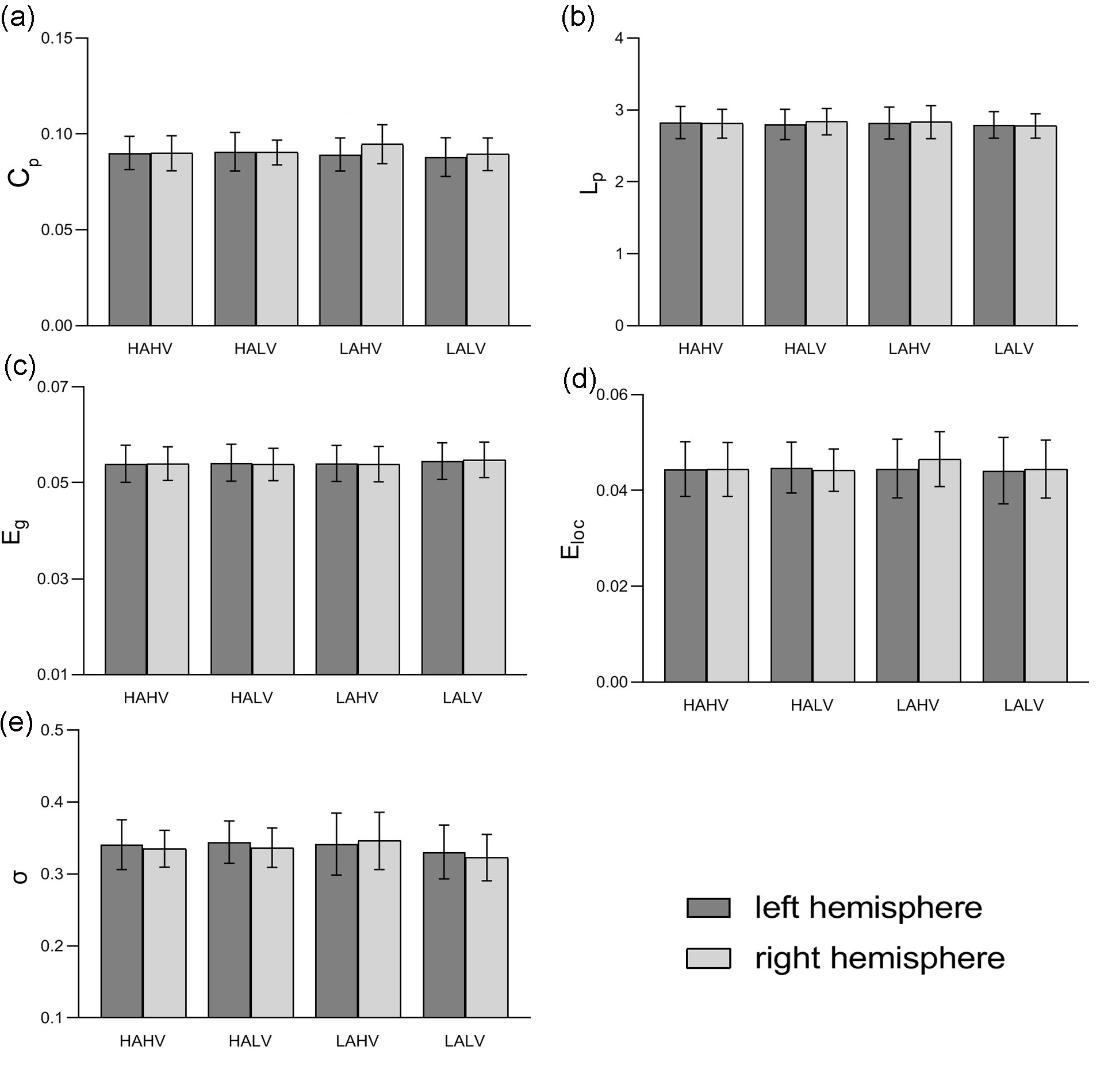

Supplement: Supplementary file 1 [file entropy-22-00939-s001.zip › Figure S2.tif]

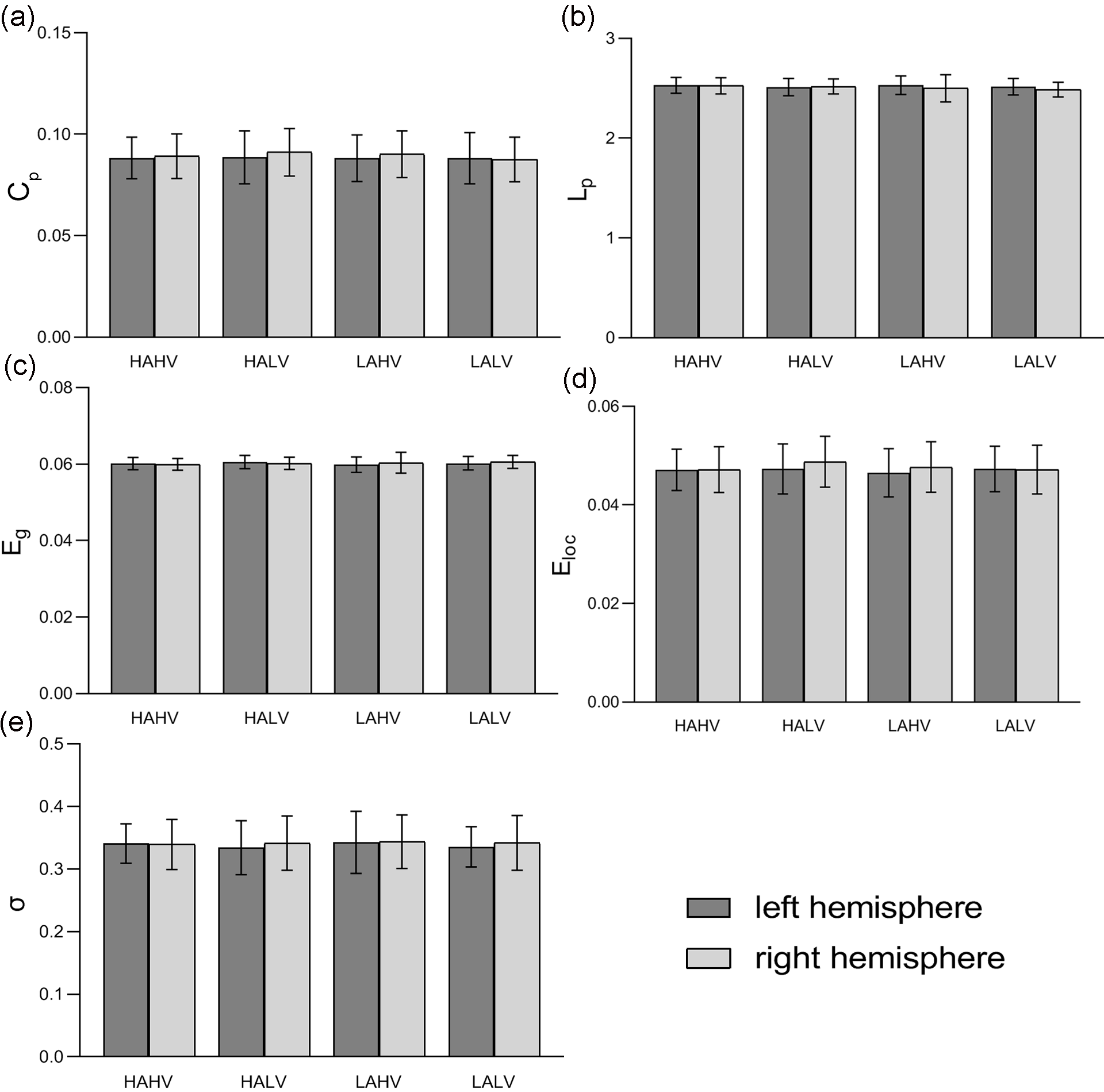

Supplement: Supplementary file 1 [file entropy-22-00939-s001.zip › Figure S3.tif]
